# Rapid Maturation of Effector T Cells in Tumors, but Not Lymphoid Organs, during Tumor Regression

**DOI:** 10.1371/journal.pone.0000821

**Published:** 2007-09-05

**Authors:** Lyse A. Norian, Paul M. Allen

**Affiliations:** Department of Pathology and Immunology, Washington University School of Medicine, St. Louis, Missouri, United States of America; New York University School of Medicine, United States of America

## Abstract

Increasing the efficacy of adoptively transferred, tumor antigen specific T cells is a major goal of immunotherapy. Clearly, a more thorough understanding of the effector phase of T cell responses, within the tumor site itself, would be beneficial. To examine this issue, we adoptively transferred tumor antigen-specific effector T cells into tumor-bearing mice, then performed kinetic evaluations of their phenotype, function, and survival in tumors, draining lymph nodes (dLNs), and spleens during regression of murine fibrosarcomas. Effector function in tumors was quantitated through the use of a novel intratumoral cytolytic assay. This approach revealed dynamic changes in the phenotype, cytolytic capacity, and viability of tumor infiltrating effector T cells during the course of tumor regression. Over a period of days, T cells within tumors rapidly transitioned from a CD25^hi^/CD27^hi^ to a CD25^low^/CD27^low^ phenotype and displayed an increase in cytolytic capacity, indicative of effector maturation. Simultaneously, however, the viability of maturing T cells within tumors diminished. In contrast, transferred T cells trafficking through lymphoid organs were much more static, as they maintained a stable phenotype, robust cytolytic activity, and high viability. Therefore, there exists a marked phenotypic and functional divergence between tumor-infiltrating effector T cells and their counterparts in lymphoid organs. Our results indicate that the population of tumor-infiltrating T cells is unique in experiencing rapid effector maturation post-transfer, and suggest that strategies aimed at prolonging the survival of CD25^low^/CD27^low^ full effectors, which displayed the highest levels of intratumoral cytolytic activity, should enhance the efficacy of T cell based tumor immunotherapies.

## Introduction

The use of T cells as a basis for immunotherapy of tumors is an area of intense investigation [1], [2]. Although much progress has been made, even the most promising clinical trials contain many patients who fail to achieve objective responses [3], and the presence of T cells in the peripheral blood or even the tumor site itself does not always correlate with tumor regression [4], [5]. This suggests that there is still much to be learned regarding the biological consequences of T cell entry into tumor microenvironments.

Tumors are known to inhibit T cell function in a variety of ways [2], [6], with many mechanisms active primarily within the confines of the tumor itself. Although tumors can exert systemic effects as well, often it is only tumor infiltrating T cells that show functional defects [7], [8], [9], due to the unique microenvironments that develop within solid tumor masses [10], [11], [12]. Although the issue of T cell function within tumors has been the focus of intense study [13]–[17], the full impact of these environments on CTL function and fate are not yet known, as it has been difficult to directly examine T cell cytolytic activity within solid tumors and most studies have focused on tumor-infiltrating lymphocyte function ex vivo. Even the use of in vivo cytotoxicity (IVC) assays, [18] which allow the quantitation of T cell cytolytic activity in spleens and lymph nodes (LNs) of tumor-bearing mice, [19], [20] does not address the impact of the tumor site itself.

In this study, we examined the intratumoral effector phase of a T cell-mediated antitumor response. We did this by monitoring potential kinetic alterations in effector T cell phenotype, function and fate as adoptively transferred effector T cells differentially trafficked through solid tumors, draining lymph nodes (dLNs) and spleens during the course of tumor regression. Our goal was to better understand how entry into the tumor site affects the capacity of T cells to bring about tumor rejection, a question that has been the subject of intense investigation [7]–[9], [13]–[17]. To provide new insight into this topic, we examined the cytolytic activity of transferred CTL in the tumor site, as well as in draining LNs and spleens. We did this by developing an assay that allowed quantitation of T cell cytolytic activity within tumors, and performed this in conjunction with IVC assays to examine kinetic differences in the functional capacity of tumor-infiltrating T cells versus their lymphoid organ counterparts.

Along with functional evaluations, we performed phenotypic assessments of transferred T cells during tumor regression. T cells are known to progress from naive to effector to memory cells over the course of weeks to months during an immune response [21], but heterogeneity exists in each of these categories of cells. For example, phenotypically distinct memory subpopulations differ in their ability to contribute to recall responses in vivo [22], [23], [24]. Effector T cells undergo phenotypic and functional conversions from early effector to full effector status [25], [26], and the ability of transferred tumor-antigen-specific T cells to bring about tumor regression was found to be dependent upon effector status at the time of adoptive transfer, with early effectors being most efficacious in vivo [26]. In several previous studies, T cell function was linked to surface expression of markers such as CD27 and CD62L, among others [24], [26]–[29]. We therefore examined whether expression levels of these proteins changed in the days following adoptive transfer of in vitro activated effector T cells, and if so, whether such changes were associated with differences in cytolytic activity.

The results described here further our understanding of effector T cell competency during an ongoing anti-tumor immune response. We found that following adoptive transfer, effector T cells infiltrating the tumor microenvironment experienced rapid phenotypic and functional alterations consistent with continued effector maturation in vivo. Within tumors, fully matured CD25^low^/CD27^low^ effector T cell populations possessed the highest level of cytolytic activity, but also the greatest incidence of apoptosis. No evidence for effector maturation was found in transferred T cell populations that trafficked through peripheral lymphoid organs. Therefore, the effector status and cytolytic capacity of tumor antigen specific T cells within tumors is dynamic, rather than constant, and changes markedly during the course of T cell mediated tumor regression. Therapies aimed at prolonging the survival of fully matured effectors should enhance the efficacy of T cell based immunotherapies. In addition, these findings illustrate the critical importance of evaluating T cell function and fate within the tumor site during the development of immunotherapeutic protocols, as highly divergent patterns of T cell phenotype, function, and fate were present in the tumor relative to peripheral lymphoid organs.

## Results

### DUC18 T cells mediate regression of CMS5 tumors

The major focus of this study was to better understand the consequences of effector T cell entry into the tumor microenvironment, with a specific emphasis on how this impacted T cell maturation and cytolytic activity. To do this, we examined the phenotype, function and fate of effector T cells that were mediating tumor rejection. To provide a context for our findings, we simultaneously examined the same parameters in T cells trafficking through lymphoid organs of these mice. We used the CMS5 fibrosarcoma model in conjunction with adoptive transfer of in vitro activated, tumor antigen-specific DUC18 T cells, because DUC18 effector T cells are readily able to infiltrate these tumors, and the ensuing tumor regression is well-characterized [30]–[33].

**Figure 1:**
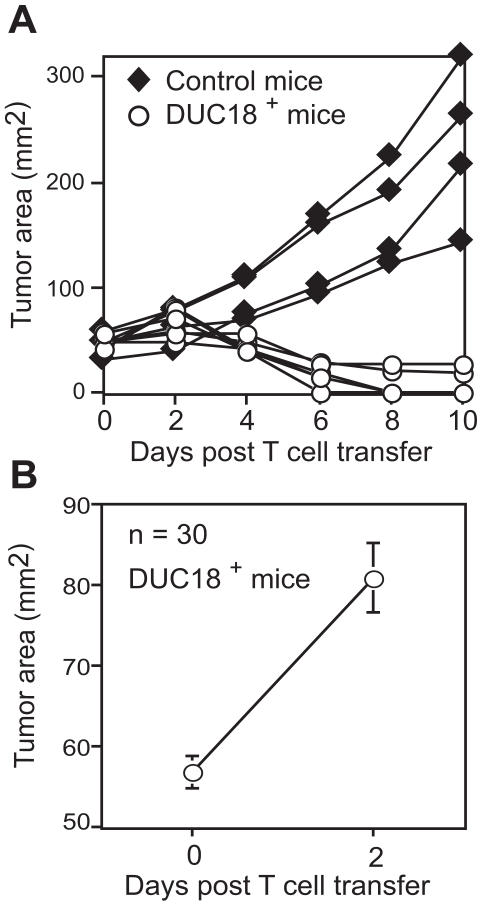
DUC18 CTL mediate regression of CMS5 tumors. 3×10^6^ CMS5 tumor cells were injected s.c. and were allowed to grow for 8 days prior to transfer of 30×10^6^ in vitro activated DUC18 T cells. This transfer protocol was repeated for all subsequent experiments. A) Tumor sizes for 6 DUC18 T cell^+^ recipient and 4 control mice are shown. Data are representative of 3 experiments. B) Mean tumor sizes +/− SEM for 30 DUC18 T cell^+^ recipient mice.

DUC18 T cells are specific for a naturally arising tumor-associated antigen antigen, denoted tERK for tumor ERK, presented by H2-K^d^ on CMS5 cells [34]. As such, CMS5 tumors act as specific targets for activated DUC18 T cells in vivo [30]–[33]. Transfer of 30×10^6^ in vitro activated DUC18 T cells caused the regression of 100% of transplanted CMS5 tumors that had grown for 8 days prior to T cell transfer (Figure 1A). The kinetics of tumor regression were very reproducible, in that tumor areas continued to increase through day 2 after T cell transfer (Figure 1B) and only declined thereafter, similar to observations in other murine tumor models [35], [36]. By focusing on these early time points, in which tumor outgrowth and subsequent regression occurred, we hoped to better understand not only how effector T cell function differed within the tumor mass relative to distal lymphoid organs, but also to identify changes that occurred in each site as the anti-tumor response progressed.

### Phenotypic maturation is unique to tumor infiltrating effector T cells

We began by evaluating the phenotype of transferred effector T cells at multiple time points during tumor regression. We wanted to determine the extent to which tumor-infiltrating T cells experienced phenotypic alterations, and also whether disparities existed between these cells and their counterparts in lymphoid organs. Both tumor draining inguinal LNs (dLNs), as a known site of tERK antigen presentation in this model [30], [31], [32], and the spleen, as an indicator of the systemic response, were examined.

We chose to examine CD25, CD27, and CD62L, as these proteins are important mediators of T cell function, and because their expression patterns had previously been shown to fluctuate as T cell effector maturation occurred [25], [26], [29]. In T cells, CD25 ligation provides signals required for optimal anti-tumor activity and CTL persistence in vivo [2], whereas CD27 regulates T cell survival [37], and CD62L mediates T cell trafficking. We therefore analyzed expression levels of these same markers on Thy1.1^+^ DUC18 T cells at the time of T cell transfer, and through day 6 post-transfer in Thy1.2^+^ recipient mice.

**Figure 2:**
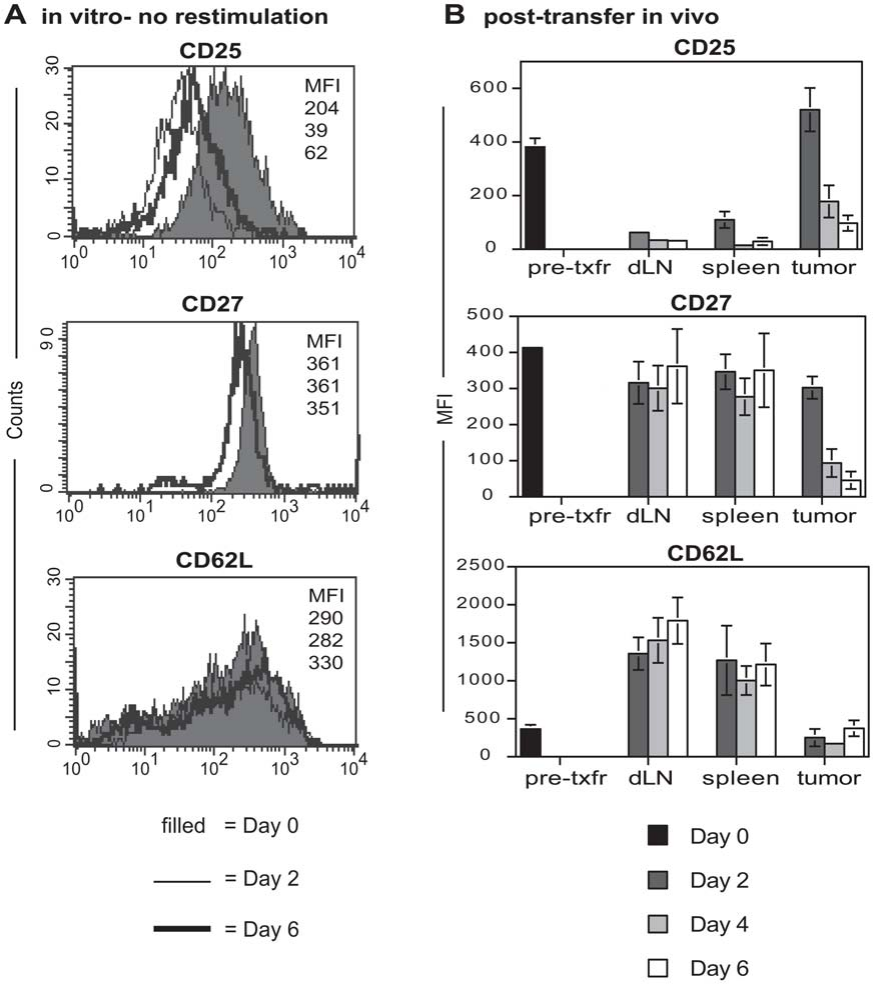
Divergent phenotypic profiles of tumor-infiltrating and peripheral lymphoid organ DUC18 CTL. Thy1.1 DUC18 T cells were activated for four days in vitro and transferred i.v. into Thy1.2 CMS5-tumor bearing mice. A) Prior to transfer, some cells were stained for CD25, CD27 and CD62L. Remaining cells were left in vitro without further stimulation, and were stained for the same markers on days 2 and 6. Data from one experiment, representative of three, are shown. B) Following DUC18 T cell transfer, dLNs, spleens, and tumors were harvested from mice and stained on days 2,4, and 6. All analyses were done by gating on live, Thy1.1^+^Vβ8.3^+^ DUC18 T cells. Shown are mean fluorescence intensity +/− SEM for combined results from 3 independent experiments, n = 8–10 mice at each time point.

In vitro activated DUC18 T cells expressed high levels of both CD25 and CD27 at the time of transfer (day 0), but expression of CD62L was variable (Figure 2A). When these cells were left in culture without further stimulation for up to 6 days, the expression levels of CD25 decreased, while CD27 and CD62L remained fairly constant. In contrast, when DUC18 effector T cells were transferred on day 0 into tumor-bearing mice, substantial changes in the expression of these markers occurred. By day 2 post-transfer, a striking phenotypic divergence was apparent between tumor-infiltrating and peripheral lymphoid organ DUC18 T cells, with regard to their CD25 and CD62L expression profiles (Figure 2B). In addition, as DUC18 CTL mediated tumor regression over days 4–6, the phenotype of effectors within tumors changed dramatically; as expected, CD25 and CD27 expression were markedly down-regulated while mean CD62L expression remained low. This phenotype is similar to that of pmel-1 CTL that have matured to a full effector state after successive rounds of in vitro stimulation [26]. In contrast, the phenotype of DUC18 T cells in the dLN and spleen remained relatively constant from days 2–6, characterized by low levels of CD25, high CD27, and high CD62L. Because the phenotype of DUC18 effector T cells maintained in vitro was distinct from either tumor-infiltrating or lymphoid organ DUC18 effector T cells, this suggests that the observed phenotypic variations in vivo occurred in response to differential signals received post-transfer, and were not the result of pre-programmed fate decisions. Therefore, within a discrete population of adoptively transferred effector T cells, phenotypic maturation occurred only in effector T cells that had infiltrated the tumor mass.

### Quantitating T cell cytolytic activity within tumors

To further our understanding of how the functional capacity of tumor-infiltrating effector T cells might vary during the course of tumor rejection, and how this might deviate from the functional capacity of effector T cells trafficking through peripheral lymphoid organs, it was necessary to examine T cell cytolytic activity within tumors. We accomplished this by developing a modification of the standard IVC assay that allowed us to quantitate target cell lysis within tumors.

**Figure 3:**
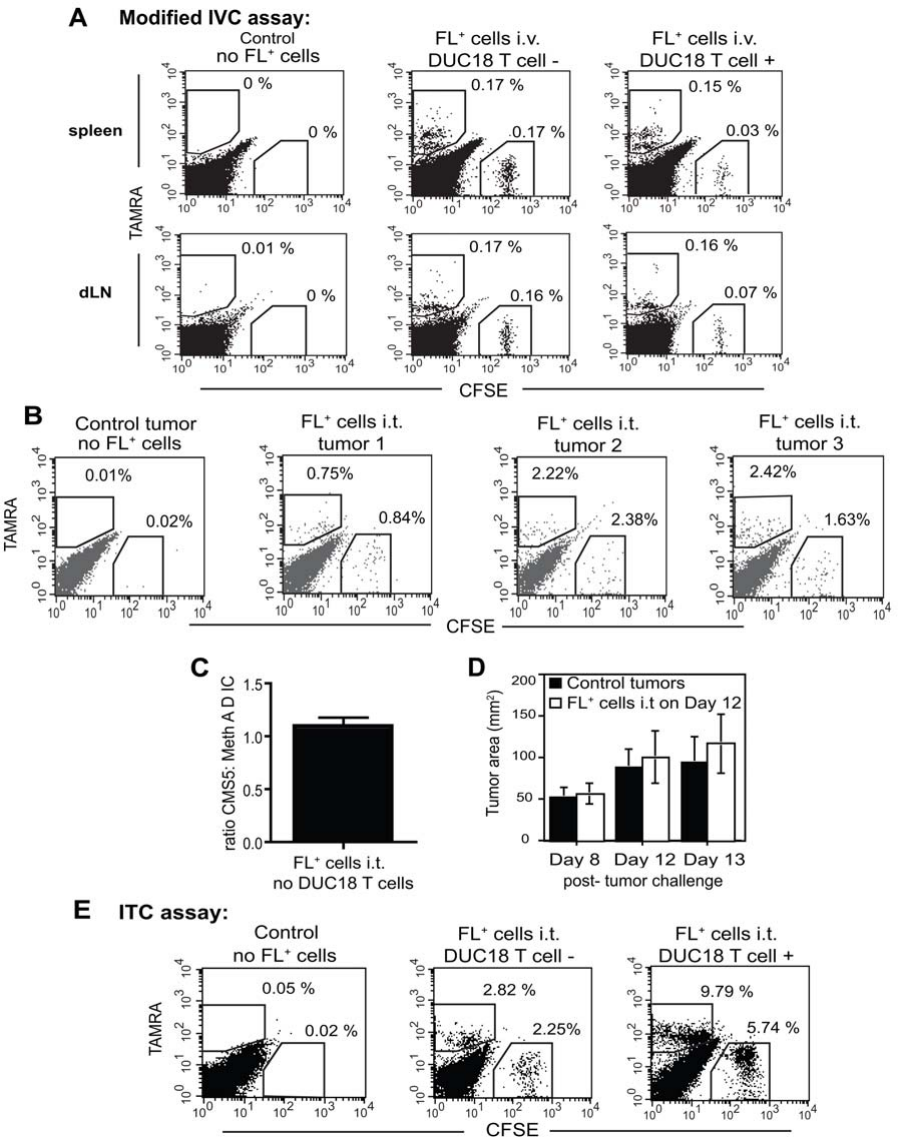
T cell cytolytic activity in peripheral lymphoid organs and tumors. DUC18 T cells were transferred as in Figure 1. A) On day 4, 5×10^6^ each TAMRA^+^ control cells and CFSE^+^ targets were injected i.v. Spleens and dLNs were harvested and analyzed on day 5. Percentages of cells within the indicated gates are shown. Data are representative of 3 experiments. B) CMS5 cells were injected s.c. and allowed to grow for 8 days. At this time, 3×10^6^ each TAMRA^+^ Meth A reference cells and CFSE^+^ CMS5 targets were injected i.t. Tumors were harvested and analyzed on day 9; the percentages of TAMRA^+^ and CFSE^+^ cells in individual tumors were determined by flow cytometry. C) The mean ratio +/− SEM of CFSE^+^ CMS5 to TAMRA^+^ Meth A cells was calculated using data from 13 individual mice. D) Tumor areas were measured on day 8 after CMS5 challenge. Four days later, fluorescently labeled tumor cells were injected as in B. Tumor sizes were measured just prior to i.t. injections and again the following day. Data represent the means +/− s.d. for 12 tumors from 4 independent experiments. No statistical difference is present in the size of control versus FL+ tumors at day 13 (p = 0.11) or in the change in control versus FL+ tumor sizes from day 12 to day 13 (p = 0.40). E) DUC18 T cells were transferred into tumor-bearing mice as in A. On day 4 post-T cell transfer, fluorescently labeled tumor cells were injected as in B. Tumors were harvested and analyzed on day 5. Percentages of cells within the indicated gates are shown. Data from 1 experiment are shown; representative of 10.

Effector T cell cytolytic activity has been examined in the spleens and LNs of mice in several model systems using a standard IVC assay with high and low concentrations of CFSE [19], [20], [38], [39]. We performed modified IVC experiments, using splenocytes pulsed with the cognate DUC18 T cell peptide, tERK, and labeled with CFSE, or splenocytes pulsed with a control nERK peptide and labeled with TAMRA prior to i.v. injection into CMS5 tumor-bearing mice. Specific loss of tERK-pulsed CFSE^+^ targets occurred only in the spleens and dLNs of mice that received DUC18 T cells (Figure 3A). These data recapitulate findings from other studies in which potent T cell cytolytic activity could be detected in spleens and lymph nodes of tumor-bearing mice [19], [20].

Our goal was to evaluate cytolytic activity of DUC18 T cells within tumors, not just spleens and LNs. Surprisingly, after i.v. injection, no labeled splenocytes were detected in any tumor harvested (data not shown). For this reason, and because we wanted to assess killing of tumor targets rather than peptide-pulsed surrogates, the protocol was further modified by using fluorescently labeled tumor cells as targets, and injecting them directly into established CMS5 tumors (intratumoral, i.t.).

For intratumoral cytotoxicity (ITC) assays, we made use of the well-described fibrosarcoma lines CMS5 and Meth A, which are antigenically distinct, yet demonstrate equivalent growth kinetics after s.c challenge in the absence of tumor antigen specific T cells, and so are often used jointly in tumor studies [32], [34], [40], [41]. CFSE labeled CMS5 cells were used as DUC18 specific targets, and in vitro comparisons demonstrated that both CFSE labeled and unlabeled CMS5 cells were killed equivalently by DUC18 effector T cells (data not shown). TAMRA labeled Meth A ΔIC cells served as reference cells, as Meth A cells do not express tERK and are not recognized by DUC18 T cells [30]. The ΔIC variant of Meth A is unable to respond to IFNγ [42] and was used to eliminate any potential loss of the reference population of fluorescently labeled cells due to bystander killing from IFNγ production directed at CMS5 targets, although parental Meth A cells were also used with similar results (data not shown). Importantly, both TAMRA^+^ Meth A ΔIC cells and CFSE^+^ CMS5 cells show equivalent viability after being injected i.t. at a 1∶1 ratio into CMS5 tumors in mice that lack DUC18 T cells (Figure 3B), although due to slight variations in the FL^+^ cell ratio in individual mice (ratios as shown: tumor 1 = 1.13, tumor 2 = 1.09, tumor 3 = 0.74), pooled tumors from multiple mice were used for subsequent experiments. When this was done, the mean ratio was calculated to be 1.095 CFSE^+^ CMS5 cells to 1.0 TAMRA^+ ^Meth A ΔIC (n = 13 independent experiments) (Figure 3C). In addition, although fairly large numbers of FL^+^ tumor cells were injected i.t., these cells routinely constituted a minor fraction of the total cell population present within solid CMS5 tumors (Figure 3B).

To address the possibility that injecting fluorescently labeled tumor cells directly into solid tumor masses might alter tumor growth, we routinely measured tumors at critical times. Analysis of multiple pooled experiments revealed no statistical differences in tumor sizes due to the i.t. injection of fluorescently labeled cells, or in the change of tumor sizes in the 24 hours following i.t. injections (Figure 3D). Thus, Meth A ΔIC cells are an appropriate reference population for use with CMS5 tumor cells, and the i.t. injection of fluorescently labeled tumor cells does not significantly alter solid tumor outgrowth during the course of the assay.

DUC18 T cell intratumoral cytolytic activity was first examined at day 4, during active tumor regression (Figure 1). In mice with no DUC18 T cells, the ratio of fluorescently labeled CMS5 to Meth A ΔIC cells again remained close to 1∶1 (Figure 3E). In DUC18 T cell recipients, the ratio of CMS5: Meth A ΔIC dropped, reflecting the specific lysis of CFSE^+^ CMS5 cells within tumors (ratio = 0.59:1). Because tumors in these mice are regressing, there is a relative increase in the percentages of fluorescently labeled cells present, as compared to progressively growing control tumors (Figure 3E). However, analyses for percent specific killing rely solely upon the ratio of CMS5 to Meth A ΔIC, so relative increases in the percentages of fluorescently labeled cells do not affect calculations or results. When similar experiments were performed in which activated DUC18 T cells were transferred into antigen-negative Meth A tumor-bearing mice, no lysis of labeled, i.t. injected CMS5 targets was observed (data not shown). This suggests that when cognate antigen is expressed on only a minority of tumor cells, insufficient numbers of effector T cells are retained in tumors to bring about detectable lysis of antigen-positive tumor targets.

### Potent CTL intratumoral cytolytic activity is preceded by a lag phase

**Figure 4:**
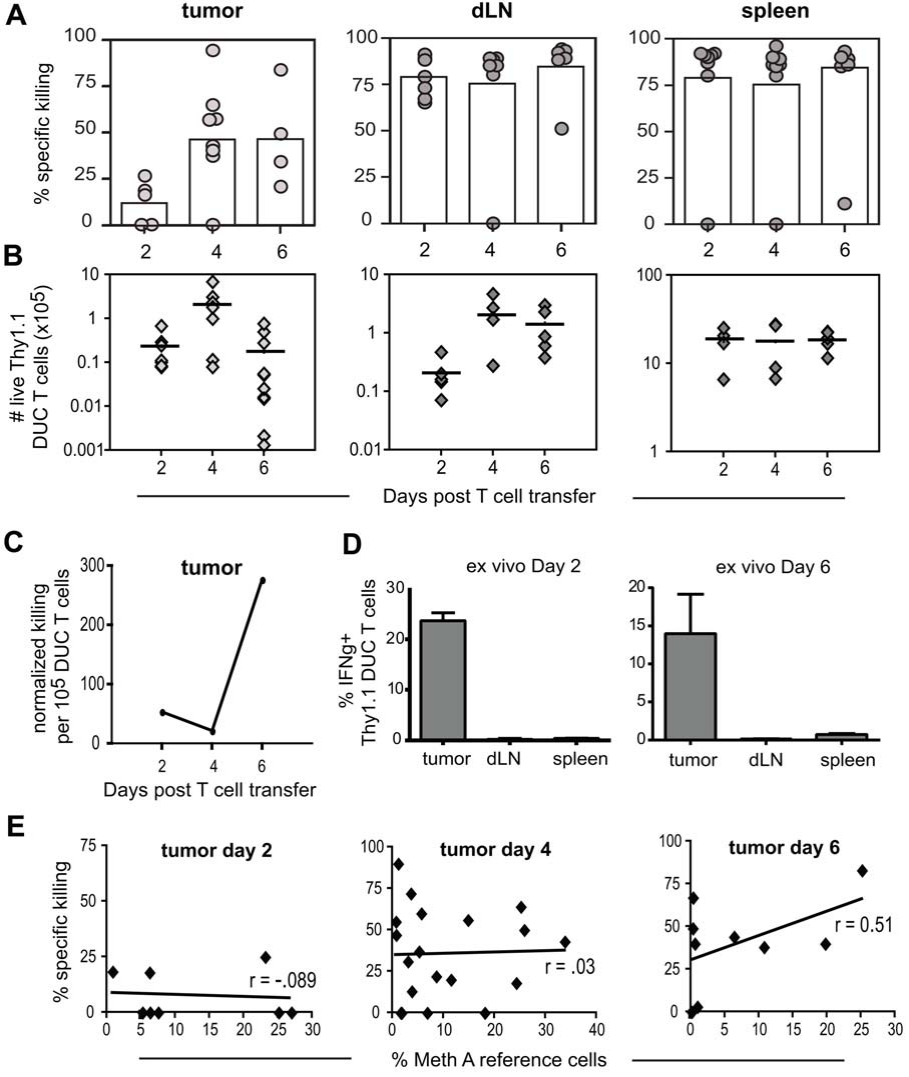
An initial lag phase in cytolytic activity precedes robust T cell mediated killing within tumors. A) ITC assays were performed beginning on days 2, 4, or 6. For tumors, each point represents the mean percent lysis, derived from 2–3 individual mice, for one experiment. Results from 17 experiments are shown, with overall mean values indicated by bars. For dLN and spleen data, each point represents killing from one mouse, with overall means indicated by bars. B) Thy1.1^+^ DUC18 T cells were transferred on day 0, and organs harvested and analyzed on indicated days. Numbers of live Thy1.1 DUC18 T cells are plotted (for tumors, p values for day 2 versus day 4 = .03, for day 2 versus day 6 = .05; for dLNs, p = 0.03 for day 2 versus day 4, and p = 0.04 for day 2 versus day 6). Points represent individual mice (n = 13–20) from 3 experiments. C) Normalized killing values were calculated by dividing the mean % specific killing by the mean number of live DUC18 T cells present in tumors for each time point. D) Activated DUC18 T cells were transferred into tumor bearing mice on day 0. Organs were harvested on days 2 and 6, and the percentage of cells expressing IFNγ ex vivo was determined by intracellular cytokine staining, after gating on Thy1.1^+^ Vβ8.3^+^ DUC18 T cells. E) Linear regression analyses for % specific killing in tumors versus % Meth A reference cells present for all individual mice used in ITC assays shown in A. The slopes were not statistically different from 0; day 2 p = 0.83, day 4 p = .90, day 6 p = .16).

After establishing a method for quantitating T cell cytolytic activity within tumors, we addressed whether cytolytic activity of tumor-infiltrating T cells increased concurrently with phenotypic maturation, and whether the kinetics of killing varied between CTL within tumors and their counterparts in peripheral lymphoid organs. For these studies, activated DUC18 T cells were transferred into CMS5 tumor bearing mice on day 0, and ITC assays were performed from days 2–6. The mean percentage of intratumoral killing is shown for each of 17 experiments (circles, Figure 4A), with overall means indicated by bars. Surprisingly, DUC18 T cell-mediated lysis of target cells was low at day 2, then increased at days 4 and 6. Modified IVC assays were performed at each time point to determine if low levels of cytolytic activity would also be detected in dLNs and spleens at day 2 or whether this trend was specific to the tumor site. In contrast to what was observed within tumors, lysis of targets by DUC18 CTL in both dLNs and spleens was high at day 2, and remained near the upper limits of quantitation throughout day 6 (Figure 4A).

Observed lysis of target cells is influenced not only by the cytolytic activity of effector T cells, but also by the numbers of live effectors present. Therefore, we tracked Thy1.1 marked DUC18 T cells after transfer into Thy1.2 recipients. The numbers of live Thy1.1^+^ DUC18 T cells within tumors increased dramatically from day 2 through day 4, but then decreased by day 6 as tumor sizes diminished (Figure 4B). This pattern varied from that observed in either dLNs or spleens of tumor-bearing mice (Figure 4B). Of note, Thy1.1^+^ DUC18 T cell numbers were comparable in tumors and dLNs at day 2, even while the amounts of target cell lysis observed in the two sites were drastically different.

To gain a sense of how the cytolytic capacity of tumor-infiltrating DUC18 CTL populations fluctuated over time, we calculated normalized killing values by dividing the mean % specific killing at each time point by the mean number of live Thy1.1 DUC18 T cells present. Doing so revealed that cytolytic capacity in tumors was low at days 2 and 4, then high at day 6 (Figure 4C), reflecting the observations that target cell lysis remained elevated even while DUC18 T cell numbers were decreasing. The cytolytic capacity of DUC18 T cells in dLNs and spleens was not evaluated, because target lysis in dLNs and spleens consistently neared the upper limits of the assay. Titrations using lower numbers of DUC18 T cells could not be performed because these conditions do not bring about tumor regression. Thus, it is possible that variations in T cell cytolytic capacity also exist in these sites.

IFNγ is often used as an indicator of effector T cell functional capacity, and it had previously been shown in another tumor model that while tumor-antigen specific effector T cells traffic systemically after adoptive transfer, they produced IFNγ only within the tumor site [43]. The data presented in Figure 4A showed that transferred DUC18 effector T cells in the dLNs and spleens of tumor-bearing mice were cytolytically active and able to mediate robust lysis of exogenous, peptide pulsed splenocyte targets. We therefore wanted to assess the functional status of effector T cells in these sites, as well as in tumors, in the absence of exogenous target cells. Intracellular IFNγ staining of Thy1.1^+^ DUC18 T cells was conducted at days 2 and 6 post-transfer into CMS5 tumor bearing mice; no exogenous target cells were given i.t. or i.v. Similar to the previous study [43], tumor-infiltrating DUC18 CTL, but not those trafficking through dLNs or spleens, were found to be producing IFNγ when examined ex vivo (Figure 4D). Therefore, although effector T cells in the dLNs and spleens are capable of lysing tERK^+^ target cells in these sites when such cells are present, in the absence of exogenous targets, these DUC18 effectors do not produce IFNγ locally.

The results presented thus far suggested that intratumoral T cell cytolytic activity increased during the course of tumor rejection. However, the ITC assay measures T cell function in a dynamic environment during tumor rejection, and it was possible that increased target cell lysis at days 4 and 6 (Figure 4A) was due to a decreasing number of unlabeled or cold competitor CMS5 tumor cells present within shrinking tumor masses. If this were true, the size of the tumor, and not the level of T cell cytolytic activity, would dictate the amount of target cell lysis observed. When the percentage of Meth A reference cells present (which would be high in small tumors, and low in larger tumors) was plotted against the percentage of specific killing detected, no positive correlation was observed (Figure 4D). Thus, heightened lysis of tumor cell targets at days 4 and 6 accurately reflects the net T cell cytolytic activity present, a product of T cell cytolytic capacity and T cell numbers, and is not spuriously affected by decreasing tumor sizes. Taken together, these results clearly show that the kinetics of killing in tumors differ greatly from what is seen in the periphery; the initial lag in observed killing was present only in tumor-infiltrating T cells.

### Diminished DUC18 T cell survival within tumors

**Figure 5:**
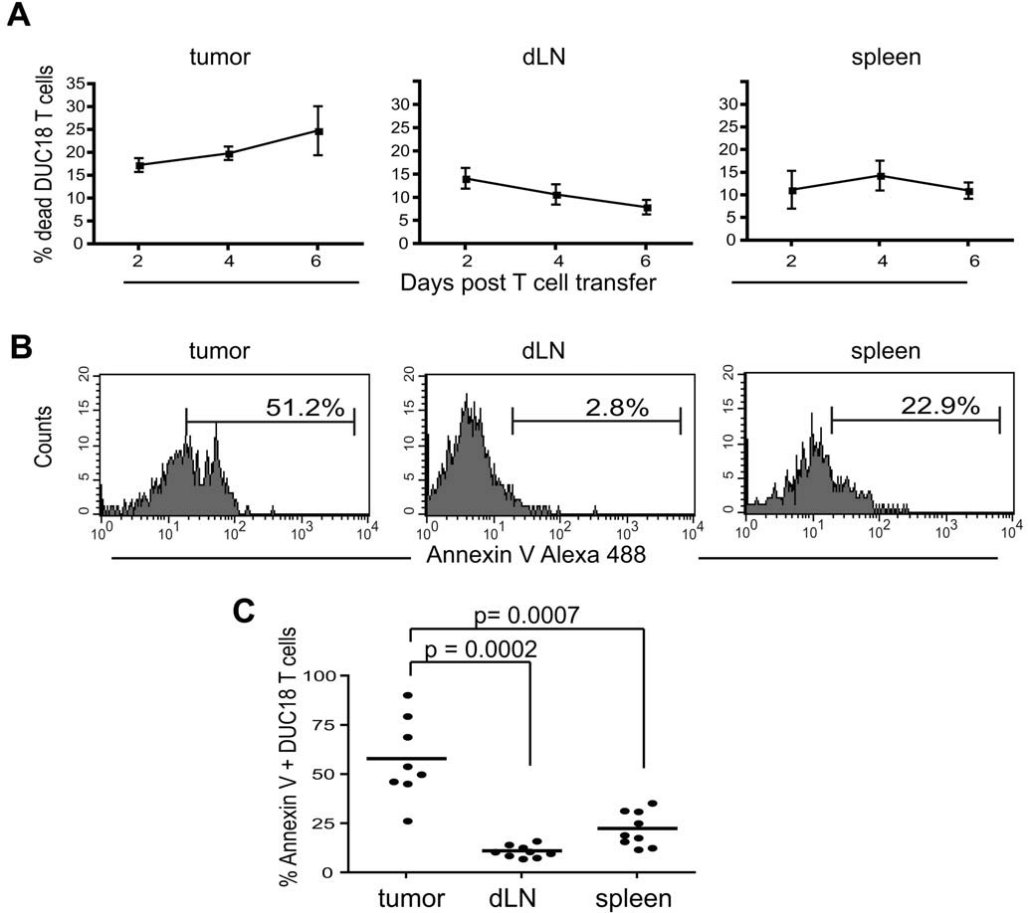
Increased cell death over time in tumor-infiltrating DUC18 CTL. Thy1.1 DUC 18 effector T cells were transferred into tumor-bearing recipients on day 0. On the indicated days, organs were harvested and stained for Thy1.1/Vβ8.3^+^ DUC18 T cells. A) The percentage of DUC18 T cells that were dead was determined by gating on the DUC18 T cell population and analyzing the PI^bright^ percentage. Data were pooled from 9 mice in 3 independent experiments. B) Organs were harvested on day 6 post-T cell transfer, and samples were stained with Thy1.1, Vβ8.3, Annexin V Alexa 488 and 7AAD. Histograms are shown after gating on the DUC18 T cell population, and represent data from a single mouse. C) The percentage of Annexin V^+^ DUC18 T cells is shown, based on the region shown in the histogram in B (p value for % apoptotic DUC18 T cells at day 6 in tumors versus dLNs = 0.0002 and for tumors versus spleens = 0.0007). Cumulative data from 3 independent experiments are shown.

Limited persistence of CTL following adoptive transfer into tumor-bearing hosts is one factor that contributes to insufficient anti-tumor immune responses, and previous studies have shown that tumor-infiltrating lymphocytes are primed to undergo apoptosis [14]. The phenotypic data presented in Figure 2 showed that tumor infiltrating T cells progressively lost expression of CD27 during the course of tumor regression, while CD27 expression on T cells in peripheral lymphoid organs remained high. Because CD27 is known to regulate T cell survival [37], [44], this suggested that effector T cells within tumors would display decreased viability. We examined DUC18 T cell viability by two methods to determine if differences would be apparent in tumor-infiltrating cells versus their lymphoid compartment counterparts. First, we compared the percentages of Thy1.1^+^ transferred T cells that were PI^bright ^in all three sites at days 2–6 after transfer. Not surprisingly, there was a slight increase in the percentage of PI^bright^ DUC18 T cells in tumors from day 2 through day 6, a trend that was not observed in dLNs and spleens (Figure 5A). A further examination of T cells at only day 6 was then done with Annexin V staining to identify apoptotic cells. Again, the highest percentages of apoptotic DUC18 T cells were found within tumors (Figure 5B) and a combined analysis of multiple experiments illustrated that this trend was consistent and statistically significant (Figure 5C). This finding is in agreement with previous reports that found fully differentiated effector T cells had impaired viability [26]. In summary, effector T cells within tumors are unique in experiencing phenotypic maturation during tumor regression, and this is associated concurrently with increased cytolytic capacity, but also decreased survival.

## Discussion

This study provides novel insight into the dynamic nature of T cell effector function in vivo, and illustrates how the effector phase of a T cell-mediated immune response is affected by local microenvironments: solid tumor versus dLN versus spleen. We found marked differences in the phenotype, kinetics of cytolytic activity, and viability of tumor antigen-specific T cells in tumors versus their counterparts in peripheral lymphoid organs, illustrating that continued effector maturation in vivo is unique to tumor-infiltrating T cells in this model system. Thus, the effector competency of tumor-infiltrating T cells can change rapidly during the course of tumor regression. Because the phenotype, function, and viability of effector T cells trafficking through peripheral lymphoid organs remained constant during tumor regression, these cells did not appear to experience in vivo effector maturation. These findings illustrate how profoundly local microenvironments affect T cell responses, and illustrate the importance of studying T cell function within solid tumors, rather than in peripheral lymphoid organs. Additionally, our findings suggest that specifically prolonging the survival of differentiated CD25^low^/CD27^low^ full effectors within tumors should enhance the efficacy of T cell mediated anti-tumor immunotherapies.

Use of the ITC assay, in conjunction with standard techniques, yielded a unique kinetic evaluation of T cell effector maturation in a population of tumor-infiltrating lymphocytes. At the time of transfer, our DUC18 CTLs possessed a defined cell phenotype and were CD25^high^/CD27^high^/CD62L^int-low^. At day 2 after transfer, cells within tumors largely maintained this phenotype, and possessed a low level of cytolytic activity. Because relatively few CTL had infiltrated the tumor mass at this point, the observed lysis of tumor targets was extremely low and correspondingly, tumor sizes had not yet begun to decline. This finding provides an explanation for the initial period of continued tumor outgrowth that follows adoptive transfer of in vitro activated, tumor antigen specific T cells in this and other murine tumor models [31], [32]. It is possible that in more highly immunosuppressive tumors, this lag phase might be extended, contributing to the failure of tumor-antigen specific T cells to control tumor growth. In the CMS5 model at day 4, the phenotype of effectors in tumors had matured, and although their cytolytic capacity remained low, the number of antigen-specific T cells had increased 10 fold. Accordingly, elevated target cell lysis was observed, and tumor regression ensued. As CTLs in tumors acquired a CD25^low^/CD27^low^/CD62L^int-low^ full effector phenotype by day 6, their cytolytic capacity increased dramatically. This was reflected in a continued diminution of tumor sizes, despite the fact that the numbers of DUC18 T cells present had declined. Similar effector maturation programs have been described previously, but in these reports, effector maturation occurred either in response to successive rounds of in vitro stimulation over a period of weeks, or over a period of weeks to months in tumor or viral infection models in vivo [26], [29], [45]. In contrast, we found that tumor infiltrating effector T cells in our model underwent a rapid phenotypic and functional conversion over a period of days, during the course of primary tumor regression.

Surprisingly, effector maturation did not occur in dLNs, even though local antigen presentation is sufficient to induce naive DUC18T cell proliferation in this model [31]. Throughout the course of tumor regression, the phenotype of DUC18 T cells in both dLNs and spleens remained CD25^low^/CD27^high^/CD62L^high^, and the viability of these cells remained high. Furthermore, when cytolytic activity was examined in dLNs and spleens of tumor-bearing mice, we observed high levels of target cell lysis even by day 2 after T cell transfer. This robust killing was present in both sites through day 6. Therefore, we observed no phenotypic or functional changes that are associated with effector maturation. It is possible that use of a lower dose of T cells, at which target cell lysis was not maximal, would show variations in cytolytic activity over time in these sites. However, lower T cell doses do not bring about consistent tumor regression in our model system, a factor that would confound results and make comparisons inappropriate. Therefore, we conclude from the IVC experiments only that T cells residing in peripheral lymphoid organs exhibited no lag phase prior to the development of robust cytolytic activity, in contrast to what occurred with tumor-infiltrating T cells. In support of this conclusion, experiments in which ITC and IVC assays were performed simultaneously in tumor-bearing mice showed that within the same animal, minimal target lysis occurred in tumors at day 2 post-T cell transfer even while high levels of target lysis were observed in dLNs and spleens (LAN, unpublished observations).

Our study suggests that there may be site-specific requirements for effector T cell maturation in order to attain protective immune responses. Previous work has shown that T cells with a full effector phenotype display the highest levels of IFNγ production and target lysis in vitro [26], and our tumor-infiltrating full effectors at day 6 possessed the highest cytolytic capacity. We speculate that within the tumor site, T cells may need to achieve a full effector status with robust cytolytic potential to overcome local inhibitory conditions. This opens the possibility that failure to achieve adequate effector maturation within the tumor microenvironment could contribute to continued tumor outgrowth in the presence of tumor antigen-specific T cells. Interestingly, effector T cells at an earlier maturational stage are able to bring about substantial cytolysis in the less hostile environments of the dLNs and spleen. Therefore, even if a linear progression of increasing cytolytic capacity is present inherently as effector T cells mature from an early to a full effector status, the effects of local microenvironments on killing of targets in vivo can not be overlooked.

At this time, many tools are available for studying T cell survival and trafficking in murine tumor models [46], and much has been learned about how to enhance anti-tumor responses in cancer patients as a result. The ITC assay described here provides a method for examining T cell cytolytic activity within the unique tumor microenvironment, and its use may shed light on ways to overcome functional deficiencies in tumor-infiltrating effector T cells. As performed in this study, the ITC assay uses antigenically distinct fibrosarcoma cell lines as the reference and target cell populations. However, many variations could be explored, such as using a single type of parental versus antigen-transfected cell line; the critical factor is that a 1∶1 ratio of target to reference cells be maintained intratumorally in the absence of antigen-specific T cells. In our hands, differentially labeling tumor target populations with CFSE did not work intratumorally, and neither did using cell lines that had been stably transfected with the fluorescent protein dsRed (data not shown). However, increasing the fluorescence intensity of reference cell population beyond what we were able to achieve would be beneficial.

Our work here provides the first quantitative evaluation of intratumoral T cell cytolytic activity. As such, it is a starting point for this type of research, and important caveats exist that should be taken into consideration. Foremost is the fact that we used transplanted fibrosarcomas; the vasculature within these tumors, and the types of stromal cells present, vary from those found in other solid tumors, such as carcinomas. Such factors can greatly influence the ability of T cells to infiltrate tumors and mediate target killing locally. Additionally, it is possible that the injection of FL^+^ cells directly into tumors could disrupt the local stroma, or induce a local inflammatory response, altering the access of T cells to their targets. However, these variables would be equally present in all tumors harvested for use in the ITC assay, and thus would not impact conclusions about the kinetics of intratumoral killing, or, for example, whether a given treatment augmented intratumoral T cell cytolytic activity. Finally, because different target cell types were used for the ITC and IVC assays, it was not possible for us to directly compare the relative levels of cytolytic activity of tumor-infiltrating T cells versus their counterparts in peripheral lymphoid organs. For technical reasons, we were unable to measure killing of labeled splenocytes within tumors, and were also unable to measure killing of labeled tumor cells in spleens or LNs after i.v. injection (data not shown). Therefore, this important issue could not be addressed using the ITC assay.

Obviously, additional studies using different T cells and tumor models will be required to test the hypotheses presented here, but continued examination of effector T cell intratumoral cytolytic activity should enhance our ability to optimize CTL function within tumor microenvironments, ultimately leading to more potent anti-tumor responses in cancer patients.

## Materials and Methods

### Mice

DUC18 TCR transgenic mice [30] are on a BALB/c background and were bred to Thy1.1 BALB/c mice (provided by Dr. Hyam Levitsky, Johns Hopkins) to generate Thy1.1^+^ DUC18 mice. BALB/c mice were purchased from the National Cancer Institute. All animal studies were performed in accordance with institutional guidelines at the Washington University School of Medicine.

### Tumor cells and in vivo challenge

The CMS5 fibrosarcoma cell line, initially derived from a BALB/c methylcholanthrene-induced fibrosarcoma [47], was cultured as described [31]. Mice were injected s.c. in the right hind flank with 3×10^6^ CMS5 cells per mouse in 200 µL of sterile PBS. Tumor outgrowth studies were conducted as described [31] with “day 0” defined as the time of DUC18 T cell transfer, eight days after tumor challenge.

### DUC18 T cell in vitro activation and adoptive transfer

Splenocytes from DUC18 mice were stimulated in vitro with one four-day round of activation [32]. Briefly, 35×10^6^ bulk splenocytes/ml were cultured with 0.5 µM tERK peptide on day 0, then were split 1∶1 on day 3 and cultured overnight without additional peptide. Dead cells were removed by Ficoll centrifugation on day 4. Prior to transfer, cells were analyzed by flow cytometry to determine the percentage of live DUC18 T cells present. For transfer, 30×10^6^ live DUC18 T cells were injected i.v. in 200 µL sterile PBS.

### Intratumoral cytotoxicity (ITC) assay

CMS5 tumor cells express the tERK/H-2K^d^ complex recognized by the DUC18 TCR, and thus serve as specific targets for DUC18 T cells [30]. Cultured cells were harvested and labeled at 1–5×10^7^ cells/ml with 5 µM CFSE (Molecular Probes) in HBSS at 37°C for 5 minutes. Cells were then centrifuged, resuspended in 100% bovine growth serum (HyClone), and incubated for 5 minutes at 37°C. MethA Δ IC fibrosarcoma cells (kindly provided by Dr. Robert Schreiber, Washington University in St. Louis) served as reference cells; they do not express tERK peptide and are not recognized by DUC18 T cells [30]. These cells are stable transfectants lacking the intracellular portion of the IFNγR [42]. Meth A ΔIC cells were cultured [42], harvested, and labeled with 12 µM TAMRA (Molecular Probes) as outlined above for CFSE. After counting, CMS5 and Meth A ΔIC were resuspended at 3×10^6^ of each cell type/50 µl of sterile PBS and injected i.t. using a 0.3cc Insulin syringe at 50 µl per mouse.

After 18 hours, tumors were harvested, homogenized, and collagenase digested [31], then filtered and resuspended in buffer (PBS with 0.5% BSA, 0.1% NaN_3_, and 10mM EDTA) for flow cytometric analysis. Typically, 250,000–500,000 events were collected, and tumors from 3 individual mice per treatment group were pooled for analysis, except where noted. Calculations for % specific intratumoral cytotoxicity were based on the equation used to calculate % specific in vivo cytotoxicity [38] such that: % killing = 100-{[(mean ratio of %CMS5 with DUC18 T cells to % MethA with DUC18 T cells)/(mean ratio of %CMS5 without DUC18 T cells to % MethA without DUC18 T cells)]×100}.

### Spleen and lymph node in vivo cytotoxicity (IVC) assays

BALB/c splenocytes were either pulsed with 0.5 µM tERK peptide (DUC18 specific targets) and labeled with 5 µM CFSE as above, or pulsed with 0.5 µM nERK peptide (a control peptide derived from native ERK2 not recognized by DUC18 T cells) [30] and labeled with 12 µM TAMRA as above. Volumes were adjusted to provide 5×10^6^ of each live cell type/200 µl sterile PBS. Suspensions were injected i.v. Approximately 18 hours later, spleens and tumor draining inguinal lymph nodes were harvested. Samples were homogenized and prepared for flow cytometric analysis as described above.

### Staining of T cells

Percentages of live Thy1.1^+^ DUC 18 T cells were determined by staining with anti-Thy1.1APC (eBioscience) and propidium iodide; some samples received anti-Vβ8.3 FITC (BD PharMingen), with anti-CD25 PE (Biolegend), CD27 PE (eBiocience) or CD62L (Biolegend). Intracellular cytokine staining was performed as described [48], using anti-Thy1.1 FITC (BD PharMingen) and anti-IFNγ APC (BioLegend). Annexin V Alexa 488 (Molecular Probes) staining was performed at room temperature in Annexin binding buffer (0.01M HEPES/NaOH pH 7.4, 0.14M NaCl, 2.5 mM CaCl_2_) for 15 minutes immediately prior to flow cytometric analysis, with dead cells being visualized by uptake of 7AAD (7 amino-actinomycin D) (Sigma).

### Statistical analyses

Determination of statistical significance was determined through use of a one or two-tailed Student's t-Test for independent samples in Microsoft Excel, or through GraphPad's Prism software for linear regression analysis.
